# Patellofemoral arthroplasty in combination with high tibial osteotomy can achieve good outcome for patients with medial-patellofemoral osteoarthritis

**DOI:** 10.3389/fsurg.2022.999208

**Published:** 2022-10-06

**Authors:** Yonggang Peng, Wei Lin, Yufeng Zhang, Fei Wang

**Affiliations:** Department of Orthopedic Surgery, Third Hospital of Hebei Medical University, Shijiazhuang, China

**Keywords:** patellofemoral arthroplasty, high tibial osteotomy, medial-patellofemoral osteoarthritis, clinical outcome, knee

## Abstract

**Background:**

The purpose of our study is to report on the clinical outcomes of patients who undergoing patellofemoral arthroplasty (PFA) in combination with a high tibial osteotomy (HTO). Due to this procedure's conservative and kinematics-preserving characteristics, we hypothesized that PFA in combination with HTO would result in good clinical outcomes in patients with medial and patellofemoral compartment osteoarthritis (MPFOA).

**Methods:**

Patients who underwent PFA in combination with HTO for MPFOA from January 2018 to April 2020 were included in the study. Clinical outcomes were analyzed by comparing the Knee Society Score, Oxford Knee Score, Range of Motion, and Forgotten Joint Score before and after the procedure. Radiological evaluations were also performed to assess the tibiofemoral osteoarthritis progression and implant loosening. For all tests, the value of *p* < 0.05 was considered statistically significant.

**Results:**

A total of nine consecutive patients who underwent PFA in combination with HTO were included. Two were males, seven were females. The average follow-up period was 2.6 ± 0.4 years. Clinical outcomes showed a significant improvement in the Knee Society Score (clinical score: 90.3 ± 8.5 and function score: 90.8 ± 7.8), Oxford Knee Score (43.6 ± 3.6), Forgotten Joint Score (71.2 ± 10.2), and knee Range of Motion (130.4 ± 8.1°) at the final follow-up. Additionally, hip–knee–ankle angle significantly decreased from −9.3 ± 2.1° preoperatively to 2.2 ± 1.2° at the final follow-up (*p *< 0.05). There were no complications for any patient during the follow-up time.

**Conclusion:**

This study shows that patients who underwent PFA in combination with HTO for the treatment of MPFOA achieved good clinical and radiological outcomes. This combined surgery could be an effective alternative to treat MPFOA in well-selected patients.

## Introduction

Osteoarthritis (OA) is the degeneration of a joint's articular cartilage and subchondral bone – results in pain and loss of function (1–3). The most commonly affected joint is the knee, and OA can affect three compartments of the knee individually or simultaneously. In fact, combined medial and patellofemoral compartment OA (MPFOA) is more common than tricompartmental disease, occurring in 23% of people undergoing primary knee arthroplasty (1). However, in clinical practice, there is still controversy about how best to manage patients with more severe patellofemoral arthritis together with (even mild) medial compartment OA, especially in relatively young patients (less than 60 years old).

Total knee arthroplasty (TKA) is one surgical option for patients with MPFOA (2, 3). However, TKA sacrifices both the cruciate ligaments and the healthy lateral compartment and leads to the disruption of the biomechanics of the knee joint (4). Another type of treatment, and one that has begun to receive renewed interest, is combining bicompartmental knee arthroplasty (BKA) implants to treat bicompartmental disease (3, 5). Compared to TKA, BKA is related to fewer perioperative complications and retains more knee function (1, 6). For patients with end-stage patellofemoral OA, patellofemoral arthroplasty (PFA) is known to be a viable solution with typically good outcomes (7–9). Unicompartmental knee arthroplasty (UKA) does well with patient satisfaction, functional outcome, and speedy recovery for patients with medial compartment OA (10). However, UKA is a technically demanding procedure with a high rate of required revision (11). Recent studies have shown that the increased failure rate of UKA is related to low-volume surgical centers and surgeons performing too few of the procedures (10, 12, 13). However, even if BKA is performed instead, the placement of the two prostheses may interfere with each other during the operation and the probability of postoperative complications such as joint stiffness is still relatively high (1, 6).

As an alternative to UKA, medial opening wedge high tibial osteotomy (HTO) has been proposed as a joint preservative, extra-articular surgery that may be a better choice for younger and more physically active patients with medial compartment OA (14, 15). Several publications have found the safety and efficacy of HTO in treating medial compartment OA in large samples (16–18).

In recent years, PFA in combination with HTO for young patients with MPFOA has been performed at our center. The purpose of this study is therefore to report the clinical outcomes in patients undergoing PFA in combination with HTO. In consideration of its conservative and kinematics-preserving characteristics, we hypothesized that PFA in combination with HTO can achieve good clinical outcomes for patients with MPFOA.

## Materials and methods

After approval from the Institutional Review Committee, a retrospective cohort study was performed on patients who underwent PFA in combination with HTO for MPFOA from January 2018 to April 2020. Inclusion criteria were as follow: (1) the presence of medial and patellofemoral OA with evident clinical symptoms (2) the presence of bone-on-bone contact at the patellofemoral joint on the skyline view (Iwano grade III-IV (19)) (3) the medial tibiofemoral OA Kellgren–Lawrence Grade III (20) (less than Kellgren–Lawrence Grade II osteoarthritis in the lateral compartment) (4) flexion contracture less than 10° (5) range of motion more than 90° (6) varus deformity less than 15° (7) the minimum follow-up time of two years. Exclusion criteria were as follow: (1) The presence of inflammatory arthritis, such as rheumatoid arthritis (2) a history of knee surgery (3) flexion contracture more than 10° (4) varus/valgus deformity more than 15° (5) range of motion less than 90° (6) anterior cruciate ligament deficiency in young patients.

### Surgical technique

All the procedures were performed by the senior surgeon using the same surgical techniques for each patient. The PFA was carried out using a standard medial parapatellar approach. The implant used in each case was a Gender Solutions PFA prosthesis (Zimmer, Warsaw, IN, USA). The first bone cut was to the anterior femoral. The anterior femoral cut was made perpendicular to Whiteside's line and parallel to the axis of the condyle. Then, a dedicated milling guide of the appropriate size was placed such that its distal end was flush with the articular cartilage both laterally and medially and its mediolateral width covered the entire trochlea. A high-velocity cutter was used to remove a minimal amount of bone and creates a bed for the prosthesis. An appropriate guide hole was done for the implant stems. The patellar was then reshaped to fit the prosthesis without resurfacing. Intraoperative assessment of patellar tracking was performed during trialing and again after cementation. When the PFA is finished, the medial opening wedge HTO was performed. A vertical skin incision was made between the anterior margin of the medial collateral ligament and the medial margin of the patellar tendon. Under fluoroscopic guidance, two Kirschner wires were then inserted into the fibular tip (approximately 1.5 cm below the joint line) from the meta-diaphyseal junction (3.5 cm–4.0 cm below the joint line), and the horizontal osteotomy was performed along the two Kirschner wires taking great care to preserve the lateral cortex. Next, an oblique coronal osteotomy procedure was performed at about 110° to the horizontal osteotomy site behind the tibial tubercle. The osteotomy was gradually opened at an appropriate angle. The side of the osteotomy was fixed with a locking plate and screws. The target mechanical tibiofemoral angle was 2°–3° of valgus. The postoperative mechanical axis was designed to pass across the knee at the Fujisawa point (a point at 62.5% of the cross-sectional diameter of the tibial plateau) (21). The same postoperative analgesia and rehabilitation protocols were used with all patients.

### Outcome evaluation

The clinical outcomes were analyzed using the Knee Society Score(KSS) (22) (including clinical and functional scores), Oxford Knee Score (OKS) (23), Forgotten Joint Score(FJS) (24), and Range of Motion(ROM) at 6 months, 1 year after the procedure then once a year. The range of motion was measured using a two-armed goniometer.

Radiological evaluations were performed based on the views of bilateral standing long-leg alignment views, standard anteroposterior, lateral view, and an axial view of the patella to assess the tibiofemoral OA progression based on Kellgren–Lawrence grade (20) and implant loosening based on the radiolucent lines of the prosthesis. The hip–knee–ankle angle (HKAA), the angle formed by the mechanical axis of the femur and mechanical axis of the tibia was recorded (25).

### Statistical analysis

All statistical analysis was performed using the SPSS software (version 23.0, IBM, Armonk, NY, USA). Data were expressed as the mean and standard deviation (SD). Differences in clinical scores were analyzed using the student's *t*-test. For all tests, a value of *p* < 0.05 was considered statistically significant.

## Results

Nine consecutive patients who underwent PFA in combination with HTO were included in the study. Two patients were males, and seven were females. The average age at the time of surgery was 57.1 ± 2.2 years. The average follow-up period was 2.6 ± 0.4 years. The average body mass index (BMI) was 25.2 ± 4.6 kg/m^2^ (Table 1).

**Table 1:** Patient demographics.

Variable	PFA + HTO
Number of patients (*n*)	9
Age, years (M ± SD)	57.1 ± 2.2
Sex, male/female	2/7
Left/Right	3/6
BMI, kg/m^2^ (M ± SD)	25.2 ± 4.6
PFOA Iwano grade III/IV (*n*)	3/6
MTFOA K-L grade II/III (*n*)	2/7
Follow-up period, years	2.6 ± 0.4

BMI, body mass index; MTFOA, medial tibiofemoral osteoarthritis; PFOA, patellofemoral osteoarthritis.

Clinical outcomes are shown in Table 2. The mean KSS clinical score significantly increased from 46.7 ± 10.3 preoperatively to 90.3 ± 8.5 at the final follow-up (*p* < 0.05), The mean KSS function score significantly increased from 43.6 ± 9.3 preoperatively to 90.8 ± 7.8 at the final follow-up (*p* < 0.05). The mean OKS score significantly increased from 19.3 ± 5.1 preoperatively to 43.6 ± 3.6 at the final follow-up (*p* < 0.05). The mean ROM significantly increased from 100.5 ± 5.8° preoperatively to 130.4 ± 8.1° at the final follow-up (*p *< 0.05). The mean of Forgotten Joint Score was 71.2 ± 10.2 at the final follow-up. Furthermore, the hip–knee–ankle (HKA) angle significantly decreased from −9.3 ± 2.1° preoperatively to 2.2 ± 1.2° at the final follow-up (*p *< 0.05).

**Table 2:** Clinical outcomes of all patient.

Variable	Preoperative	Last follow-up	*P* value
KSS clinical score	46.7 ± 10.3	90.3 ± 8.5	<0.05
KSS function score	43.6 ± 9.3	90.8 ± 7.8	<0.05
OKS	19.3 ± 5.1	43.6 ± 3.6	<0.05
ROM (°)	100.5 ± 5.8	130.4 ± 8.1	<0.05
HKAA (°)	−9.3 ± 2.1	2.2 ± 1.2	<0.05
FJS		71.2 ± 10.2	

KSS, knee society score; OKS, oxford knee score; FJS, forgotten joint score; ROM, range of motion; HKAA, the hip–knee–ankle angle, positive represents valgus, negative represents varus.

**Figure 1:**
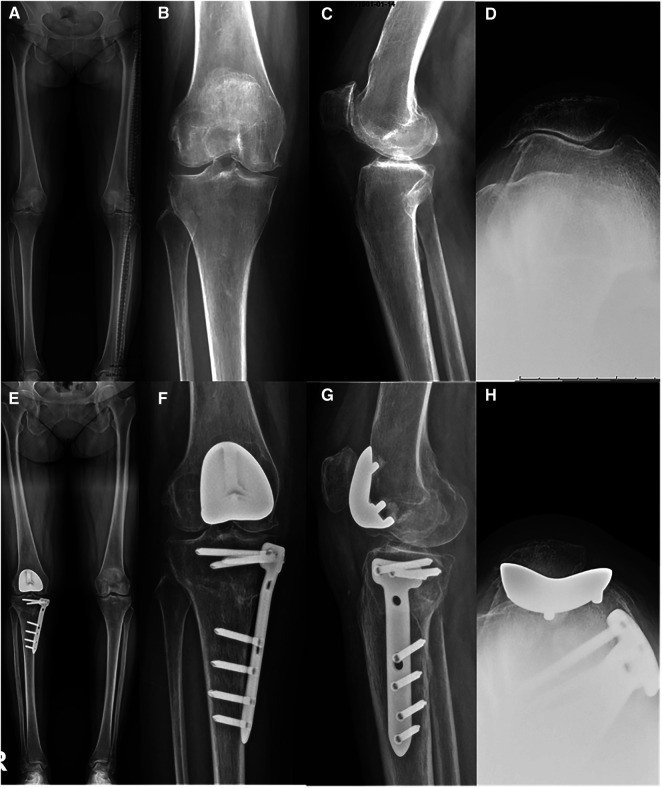
X-rays of a patient with the patellofemoral arthroplasty in combination with high tibial osteotomy preoperative (**A–D**) and postoperative at six months (**E–H**).

There were no complications such as dislocation, patellar maltracking, patellofemoral squeaking, or infection, and there were no signs of osteolysis or subsidence during the follow-up period. The x-ray of one patient is shown in Figure 1.

## Discussion

The most important finding of this study is that patients who underwent PFA in combination with HTO had a significant improvement in KSS, OKS, and ROM, and achieved a significantly high FJS, showing that patients experienced significant improvements in terms of pain and knee function at a mean follow-up of 2.6 years.

During clinical practice, the question of how to treat patients with MPFOA is still somewhat controversial. Although TKA remains a successful treatment choice for patients with severe MPFOA (2, 3, 26), TKA is not ideal for younger people who are employed and/or active (27, 28). Because TKA sacrifices the cruciate ligaments and the healthy lateral compartment. TKA could also lead to disturbed biomechanics over the knee joint and wastes valuable fallback positions in case of failure (29).

For patients with MPFOA, some authors have advocated bicompartmental knee arthroplasty (BKA) as a solution, since BKA can preserve the healthy compartments of the knee and the cruciate ligament that are essential for physiological tibiofemoral kinematics and maintenance of proprioception (30, 31). Advocates of BKA have stated that its potential advantages include less blood loss, faster return to normal activity, shorter hospital stay, higher stability, and less pain (2, 32). But, opponents have pointed out that these advantages have not been shown to be sustained after 1 year postoperatively and that they are in fact minimal after adjusting for age, sex, BMI, and baseline status (33). BKA is also more technically demanding, resulting in increased operation duration (3). Overall revision rates of UKA are high (11). The failure rate of UKA may be due to low-volume surgery centers and surgeons performing a lower number of such procedures in general (10, 12, 13).

In this study, the mean age of the patients was 57 years old. Based on the disadvantages of TKA, we thought that TKA was not a good choice. For patients with severe patellofemoral OA and moderate medial compartment OA, especially those with varus deformity, BKA maybe not be suitable for them wither. As a result, we chose PFA combined with HTO for them.

PFA has been received as a less invasive alternative to TKA for patients with isolated patellofemoral OA (9, 34, 35). Compared to TKA, PFA provides more bone conservation, reduced blood loss, shorter operation times, shorter post-operative rehabilitation periods, and more functional knee in the younger, active patients (34, 36). Odgaard, A et al. demonstrated that patients who received PFA achieved a better knee-specific quality of life than those who received TKA during the first 2 years. Patients receiving PFA have been shown to regain their preoperative ROM, whereas patients receiving TKA have been found to lose 10° of ROM two years postoperatively (36). Furthermore, a systematic review has shown that the survival rate of PFA is 92% at more than 5 years of follow-up (37). Thus, PFA can indeed be considered a good approach for patients with isolated PFOA.

In conjunction with PFA, HTO is used to rearrange the mechanical axis of the lower limb to transfer weight-bearing areas to nonaffected areas (38). In this way, the damaged cartilage of the knee can be unloaded, thereby reducing pain, improving function, slowing knee deterioration, and possibly delaying the need for arthroplasty (39). HTO is a joint-preserving procedure that does not compromise future TKA (16). For young patients with higher physical demands, such as participation in sports or employment, high tibial osteotomy (HTO) is therefore superior to arthroplasty in the treatment of unicompartmental OA (27, 28, 40). Several studies have demonstrated the advantages of HTO, including a study of 79 knees treated with HTO in which the survival rate was 81.7% at 10 years (41). Thus, HTO may be more suitable for younger patients with higher physical demands.

What's more, however, is that PFA combined with HTO permits the preservation of the cruciate ligaments and requires minimal bone excision, resulting in rapid recovery to normal activity as well as decreased pain. Studies have shown that maintaining the anterior cruciate ligament can be beneficial for joint kinematics, the ability to climb stairs, and patient satisfaction (42). One study reported that six knees underwent the inlay trochlear implant resurfacing and HTO in middle-aged athletes (43). The patients achieved good outcomes. Our results have shown that PFA in combination with HTO was a successful surgical treatment option for patients with MPFOA (less than 60 years old). In addition to KSS, and OKS, we also used FJS to evaluate patients' postoperative status. FJS can measure a patients' ability to forget joint awareness in daily life. Lin et al. reported that patients who underwent PFA had a significantly higher FJS than those who underwent TKA (44). The average FJS was 71.2 ± 10.2 in our study at the last follow-up, indicating that the patient had been able to forget joint awareness.

Limitations of the present study include its retrospective nature, its lack of a matched cohort treated with TKA, and its relatively small sample size. However, these results demonstrate that PFA in combination with HTO is a reasonable choice for the treatment of MPFOA in the middle term. A longer follow-up will ultimately be required to evaluate the long-term outcomes. In addition, comparative studies need to be performed to compare TKA and PFA in combination with HTO in the treatment of MPFOA.

## Conclusion

This study showed that the patients who underwent PFA in combination with HTO for the treatment of MPFOA achieved good clinical and radiological outcomes. This combined surgery could be an effective alternative to treat MPFOA in well-selected patients.

## Data Availability

The raw data supporting the conclusions of this article will be made available by the authors, without undue reservation.
